# Dexamethasone in Glioblastoma Multiforme Therapy: Mechanisms and Controversies

**DOI:** 10.3389/fnmol.2019.00065

**Published:** 2019-03-29

**Authors:** Marta Cenciarini, Mario Valentino, Silvia Belia, Luigi Sforna, Paolo Rosa, Simona Ronchetti, Maria Cristina D’Adamo, Mauro Pessia

**Affiliations:** ^1^Section of Physiology and Biochemistry, Department of Experimental Medicine, University of Perugia School of Medicine, Perugia, Italy; ^2^Department of Physiology and Biochemistry, Faculty of Medicine and Surgery, University of Malta, Msida, Malta; ^3^Department of Chemistry, Biology and Biotechnology, University of Perugia, Perugia, Italy; ^4^Department of Medical-Surgical Sciences and Biotechnologies, University of Rome “Sapienza”, Polo Pontino, Latina, Italy; ^5^Section of Pharmacology, Department of Medicine, University of Perugia School of Medicine, Perugia, Italy

**Keywords:** GBM, dexamethasone, cerebral oedema, pharmacogenomics, glioblastoma multiforme therapy

## Abstract

Glioblastoma multiforme (GBM) is the most common and malignant of the glial tumors. The world-wide estimates of new cases and deaths annually are remarkable, making GBM a crucial public health issue. Despite the combination of radical surgery, radio and chemotherapy prognosis is extremely poor (median survival is approximately 1 year). Thus, current therapeutic interventions are highly unsatisfactory. For many years, GBM-induced brain oedema and inflammation have been widely treated with dexamethasone (DEX), a synthetic glucocorticoid (GC). A number of studies have reported that DEX also inhibits GBM cell proliferation and migration. Nevertheless, recent controversial results provided by different laboratories have challenged the widely accepted dogma concerning DEX therapy for GBM. Here, we have reviewed the main clinical features and genetic and epigenetic abnormalities underlying GBM. Finally, we analyzed current notions and concerns related to DEX effects on cerebral oedema, cancer cell proliferation and migration and clinical outcome.

## Introduction

Glioblastoma multiforme (GBM) is the most aggressive form of brain tumor, accounting for 54% of all gliomas (Dolecek et al., 2012). The estimated number of annual new cases of GBM are: Japan 2,200, UK 2,531, France 3,000, Germany 3,500, USA 18,000. Globally, over 100,000 patients die each year as a result of this type of brain cancer. It is highly invasive and not amenable to current treatment such as surgical management, chemo- and radio-therapy (Furnari et al., 2007) and affected individuals have very poor life expectancy (Miller and Perry, 2007). The highly aggressive course and poor response to treatments likely result from uncontrolled cellular proliferation, migration, presence of a cancer stem-like cell population, neo-angiogenesis and severe brain oedema.

GBM-induced cerebral oedema is currently treated with corticosteroids due to their ability to decrease the permeability of the blood brain barrier (BBB; Salvador et al., 2014). Dexamethasone (DEX) represents the drug of choice among the synthetic glucocorticoids (GCs) by virtue of its minimal mineralocorticoid activity, long half-life and high potency (Kostaras et al., 2014). By evaluating the typical features of GBM aggressiveness, several studies have shown positive effects of DEX on GBM cells both *in vitro* and cancer volume, *in vivo* (Guerin et al., 1992; Wolff et al., 1997; Kaup et al., 2001; Villeneuve et al., 2008; Piette et al., 2009; Fan et al., 2014) although, the mechanisms accounting for these actions remain unclear. Strikingly, contradictory evidence has been published generating controversies that are still debated. As a resource for those interested in this issue, we have reviewed relevant critical articles from various research groups describing DEX actions in GBM and discussed proposed mechanisms and controversies.

## Glioblastoma Multiforme: Clinical and Molecular Features

GBM is known as grade IV astrocytoma according to the World Health Organization (WHO) classification. “Primary GBM” accounts for some 90% of cases, arises *de novo* and affects older patients. “Secondary GBM” develops from preceding low-grade astrocytomas and is more common in patients that are ≤45 years old (Crespo et al., 2015; Louis et al., 2016). Neurological signs depend on the location of the tumor within the brain and can be either focal or generalized. Frequently, symptoms include headache, seizures, cognitive dysfunction, ataxia, vomiting, vision disturbance. Unusual symptoms such as syncope, vertigo, hypoesthesia or psychiatric manifestations could result in misdiagnosis and mistreatment. In some instances, GBM infiltrate the brain as finger-like tentacles making surgical resection of the tumor very difficult, especially when it develops near eloquent regions of the brain. GBM could be considered a non-metastatic cancer, as it rarely spreads from the primary site to other remote tissues of the body thanks to the confining properties of the BBB (Beauchesne, 2011). Another typical feature is the presence of hypoxic and necrotic regions within the cancer mass (Lara-Velazquez et al., 2017). Microscopic analyses showed a central large necrotic core that takes up to 80% of tumor mass and multiple thrombotic foci surrounded by pseudopalisading cells migrating towards more oxygenated areas (Rong et al., 2006; Amberger-Murphy, 2009; Sforna et al., 2017). Hypoxia induces important changes on tumor cell genome and proteome, which increase its aggressiveness (Amberger-Murphy, 2009). In particular, hypoxia promotes invasiveness, radio- and chemo-resistance, glioma stem cell (GSC) development and angiogenesis (Jensen, 2009; Yang et al., 2012; Sforna et al., 2015). The vascular endothelial growth factor (VEGF) is over-expressed by cancer cells under hypoxia and promotes neo-angiogenesis (Soda et al., 2013; Dubois et al., 2014). Indeed, GBM is a highly vascularized tumor and the degree of vascularization influences prognosis, remarkably (Takano et al., 2010; Dubois et al., 2014).

Histological evaluations have shown extensive variability of cell morphology that is characterized by the coexistence of small cells and multinucleated giant cells (Meyer-Puttlitz et al., 1997; Kleihues, 1998). A growing body of evidence supports the notion that GBM cell heterogeneity results also from the presence of GSCs, a small subpopulation of tumor cells that show features of self-renewal, resistance to radiotherapy (RT) and chemotherapy, ability to differentiate *in vitro* and promote tumor recurrence. These properties are shared in part with neuronal stem cells (NSCs; Reya et al., 2001; Bao et al., 2006; Vescovi et al., 2006; Park and Rich, 2009; Rosen and Jordan, 2009; Frank et al., 2010; Heddleston et al., 2010). Of note is that, GSCs are also responsible for the angiogenic potential of the tumor by expressing high levels of VEGF (Bao et al., 2006; Li et al., 2009). Initially, the term multiforme referred to a broad spectrum in cellular heterogeneity. However, in addition to the histological features, GBM is characterized by several genetic and epigenetic alterations. Thus, multiforme currently refers to the latter multifaceted features of the cancer (DeAngelis and Mellinghoff, 2011; Stoyanov et al., 2018). Indeed, genomic investigations resulted in the identification of a number of genetic abnormalities underlying the molecular transcriptional subtypes of GBM. Verhaak et al. (2010) proposed the classification of GBM into four distinct subtypes: classical, mesenchymal, proneural and neural.

The classical subtype is characterized by amplification or mutation in the *epidermal growth factor receptor* (EGFR) and deletion mutations in the *cyclin-dependent kinase inhibitor 2A* (*CDKN2A*) gene, coding for the p16INK4A and p14arf protein tumor suppressors. Genomic amplification of EGFR is observed in 97% of the classical subtype, which leads to the four-fold increase in EGFR expression. The most common point mutation results in the deletion variant EGFRvIII, lacking exons 2–7, which encode the extracellular domain. The EGFRvIII alteration prevents EGF binding and confers ligand-independent signaling, thereby activating pathways other than those controlled by the wild-type EGFR.

The mesenchymal subtype is featured by deletions of a region at 17q11.2 containing the gene NF1 (*neurofibromin*). Moreover, genes in the *tumor necrosis factor* (TNF) super family pathway are highly expressed in this subtype, potentially as a consequence of higher overall necrosis (Verhaak et al., 2010; DeAngelis and Mellinghoff, 2011; Lara-Velazquez et al., 2017).

The proneural subtype is characterized by high levels of *platelet-derived growth factor receptor A* (PDGFRA) expression and point mutations of both *isocitrate dehydrogenase 1* (IDH1) and p53. Although, focal amplifications of the locus at 4q12 harboring the PDGFRA gene were observed in all GBM subtypes, the proneural samples possess a much higher degree. Multiple PDGFRA point mutations have been identified in the Ig-domain, potentially disrupting ligand interaction. Moreover, a rare in frame deletion of the Ig-domain of PDGFRA has been described (Kumabe et al., 1992; Rand et al., 2005). The IDH family includes three enzymes with different locations: IDH1 found in cytosol and peroxisome and IDH2 and IDH3 located in mitochondria. These are involved in the biosynthesis of central metabolites in the tricarboxylic acid (TCA) cycle and catalyze the oxidative decarboxylation of isocitrate to α-ketoglutarate (α-KG), producing NADPH (Leonardi et al., 2012; Miller and Perry, 2007). The proneural subtype is also characterized by point mutations in the IDH1 gene. These mutations produce d-2-hydroxyglutarate (d-2HG), a competitive inhibitor of alpha-ketoglutarate-dependent dioxygenase which induces epigenetic changes, including hypermethylation (Waitkus et al., 2016; Czapski et al., 2018; Lee et al., 2018).

The neural subtype is characterized by expression of neural markers such as *neurofilament light* (NEFL), *gamma-aminobutyric acid type A receptor alpha-1 subunit* (GABRA1) and the SLC12A5 (*K ^+^/Cl^−^ co-transporter 2*).

According to recent WHO guidelines, GBMs could be subdivided into IDH wild-type and IDH-mutant (Louis et al., 2016). GBM/IDH wild-type are more common (~90%), tend to be more aggressive, and have worse prognosis than GBM/IDH mutant. Mutations in the IDH-encoding gene have been found in ~10% of GBM (Parsons et al., 2008) and are associated with altered cell metabolism. IDH mutations were mainly found in secondary GBM, which develops from low-grade gliomas (Han and Batchelor, 2017).

IDH1 mutations occur in 50%–80% of grade II and III astrocytoma, oligodendroglioma and secondary GBM. IDH2 mutations occur in the same tumor with less frequency (Hartmann et al., 2009). The most common mutation in IDH1 results in arginine 132 to histidine substitution (R132H). This mutation converts α-KG to the R(−)-2-hydroxyglutarate (2-HG), considered as a potential “*oncometabolite*.” It has been shown that accumulation of 2HG affects histone and DNA demethylases resulting in a hypermethylation phenotype with chromatin modifications and gene expression dysregulation (Staedtke et al., 2016; Han and Batchelor, 2017).

### The Epigenetic Origin of GBM and Therapeutic Potential of Epigenetic Modifiers Synergized by DEX

Epigenetic modifications refer to changes in gene expression and cellular phenotype without alterations in the DNA sequence. A number of studies over the past years have provided greater knowledge and insight concerning the epigenetic origin of GBM. *Hypermethylation* represents an epigenetic mechanism frequently observed in GBM that occurs at genes involved in cell cycle regulation, DNA repair, apoptosis, angiogenesis and invasion (Alaminos et al., 2005; Tews et al., 2007; Martinez and Esteller, 2010). DNA hypermethylation of promoter regions can silence tumor suppressor genes or pro-apoptotic genes, or even favor the response to chemotherapy and RT in tumor cells (Kanazawa et al., 2019). Remarkably, 90% of high-grade gliomas contained methylated gene promoters. Affected individuals were found to have large amounts of DNA in the plasma and the same methylated promoters present in the tumor were also found in the plasma in 60% of the cases. This represents the first step towards the development of quantitative plasma biomarkers that could be used to monitor glioma status (Weaver et al., 2006). Methylation of the *O6-methylguanine DNA methyltransferase* (MGMT) promoter is frequently observed in secondary GBM, associated with p53 mutations (Nakamura et al., 2001; Martinez and Esteller, 2010). MGMT is involved in DNA repair and its activity is down-regulated by methylation.

Methylation, demethylation and acetylation are among the main epigenetic modifications of histone. Histone methylation and demethylation are involved in reprogramming GBM cell metabolism and occur by action of histone methyltransferases (HMTs) and demethylases (HDMs), respectively (Dong and Cui, 2018). Histone acetyltransferases (HATs) and deacetylases (HDACs) control the acetylation state of histones, modulating chromatin structure and function and promote the transcriptional activation or repression (Bezecny, 2014). HDACs also change non-histone proteins which regulate important cellular functions such as cell-cycle progression, differentiation and apoptosis (Lee et al., 2015). The HDAC family includes four classes: Zn^2+^-dependent (classes I, II and IV), Zn^2+^-independent (class III) and nicotinamide-adenine dinucleotide-dependent enzymes (Lee et al., 2017). In GBM, HDACs are considered the main effectors of epigenetic alterations and are implicated in tumorigenesis. Indeed, mutations and alterations of HDAC expression have been identified and associated to GBM pathogenesis and progression (Was et al., 2019). In particular, mutations in both HDAC2 and HDAC9 genes have been found through sequencing of GBM biopsies (Parsons et al., 2008). It has been reported that GBM displays decreased mRNA expression in class II and class IV HDACs. On the other hand, recent studies have shown increased levels of HDAC1, HDAC3, and HDAC6 (Staberg et al., 2017), as well as overexpression of HDAC9 (Yang, 2015).

The reversibility of epigenetic modifications opens new therapeutic perspectives for GBM. As such, the search for new drugs targeting epigenetic modifications has been expanded over the past years. DNA methyltransferase inhibitors (DNMTi) and histone deacetylase inhibitors (HDACi) have been tested in multiple cancers. However, only HDACi have been approved in clinical trials (Romani et al., 2018). HDACi are divided into seven categories: short chain fatty acids, benzamides, cyclic peptides, electrophilic ketones, hydro-xamines, sirtuin inhibitors and other miscellaneous forms (Lee et al., 2015). Their anti-cancer effects include the induction of cell-cycle arrest, differentiation, apoptosis, mitotic cell death and autophagic cell death (Bezecny, 2014). *Vorinostat*, *Romidepsin* and *Valproic Acid* are among the HDCAi that managed to find their way in clinical trials. The efficacy of *Vorinostat* has been tested in primary GBM explants, as well as in murine GBM cell lines, *in vitro* and *in vivo*. The drug induced accumulation of cells in the G2-M phase, increased the expression of p21WAF1, p27KIP1, DR5 and TNFα, and decreased the levels of the pro-growth genes CDK2, CDK4, cyclin D1 and cyclin D2. In addition, it reduced the invasiveness in a number of GBM cell lines with different PTEN and p53 mutations (An et al., 2010; Xu et al., 2011). *Romidepsin* is a class I HDACi and exerts its functions by down-regulating Bcl-xL and up-regulating p21 expression (Lee et al., 2015).

The *Enhancer of Zeste Homolog 2* (EZH2) is a histone-lysine N-methyltransferase enzyme that participates in histone methylation. EZH2 is the functional enzymatic component of the *Polycomb Repressive Complex 2* (PRC2) involved in a wide range of glioma processes, including cell cycle, invasion, GSC maintenance, drug and RT resistance (Yin et al., 2016). A recent study showed the effects of the SAM-competitive EZH2 inhibitor UNC1999 in different stem cell-like glioma cells, named brain tumor-initiating cells (BTICs). By evaluating cell growth, the combination of UNC1999 with DEX exerted a synergistic effect in two different BTICs type and suppressed tumor growth, *in vivo* (Grinshtein et al., 2016). Therefore, therapeutic anti-cancer benefits may result from the combination of drugs which enhance therapeutic efficacy compared to the mono-therapy approach derived through targeting key pathways in a characteristically synergistic or additive manner. However, a common obstacle shared by all these therapeutic approaches is the low permeability of the BBB. It is hoped that the use of easily penetrating drugs, such as DEX, together with newly selected epigenetic compounds that are able to cross the BBB could contribute to finding a better therapy for GBM.

## GBM-Induced Cerebral Oedema

Cerebral oedema is a hallmark of malignant brain tumors that influences the clinical course and the prognosis of the disease (Stummer, 2007; Lin et al., 2016). It represents a major cause of morbidity and mortality in GBM due to the high risk of brain herniation in up to 60% of patients (Silbergeld et al., 1991). Indeed, the accumulation of fluids inside the cerebral parenchyma induces a rapid increase in brain volume leading to a sharp increase in intracranial pressure (ICP), which may cause ischemia, herniation and ultimately death (Papadopoulos et al., 2004). The main processes involved in cerebral oedema are vasogenic and cytotoxic oedema. Vasogenic oedema results from accumulation of fluid in the cerebral parenchyma caused by disruption and increased permeability of the BBB. Cytotoxic oedema results from failure in cell metabolism leading to impairment of the Na^+^/K^+^ pump (Michinaga and Koyama, 2015). In gray and white matter, astrocytes may swell as a result of cytoplasmic retention of Na^+^ ions and water. Typically, GBM-associated cerebral oedema is vasogenic in nature. It is characterized by breakdown of the BBB, resulting in extracellular accumulation of fluid with disruption of homeostasis around the microenvironment of the compromised parenchyma.

**Figure 1:**
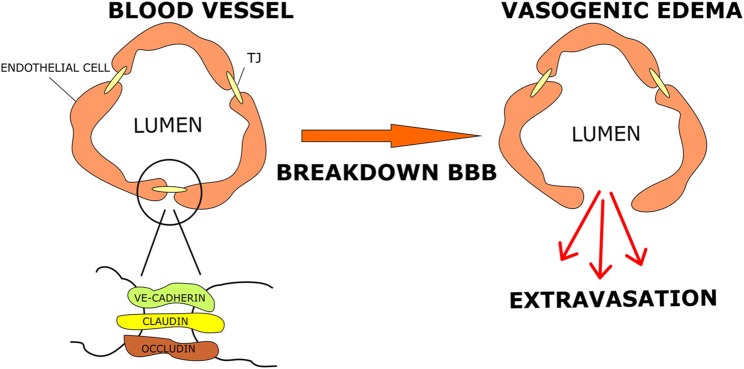
Tumor-related oedema. Brain capillary endothelial cells (BCECs) are connected *via* tight junction (TJ) protein complexes that fuse endothelial cells together. When a brain tumor develops, particularly high-grade gliomas, TJs become permeable due to release of angiogenic factors and changes in protein component. These events cause blood brain barrier (BBB) breakdown and vasogenic oedema.

The BBB represents a structure that separates the brain parenchyma from the circulatory system, allowing maintenance of central nervous system (CNS) fluid homeostasis and passage of substances to brain cells by mechanisms that rely on simple diffusion or active transport (Campos-Bedolla et al., 2014). It is formed by brain capillary endothelial cells (BCECs) that are supported by neighboring glial cells (microglia and astrocytes; Kaur and Ling, 2008), neurons and perivascular pericytes (Wolburg and Lippoldt, 2002; Zlokovic, 2008; Zozulya et al., 2008). The BCECs are joined together by means of tight junctions (TJs), composed of several proteins, among which are claudins and occludins (Furuse et al., 1993, 1996, 1998; Kubota et al., 1999). These transmembrane proteins bind intracellular proteins such as zonula occludens-1 and -2 (ZO1; ZO2) allowing the coupling of TJs to the cytoskeleton elements of endothelial cells (Stummer, 2007). The TJ formation is promoted by growth factors secreted by astrocytes which therefore play an important role in controlling the unique tightness of the BBB (Wolburg et al., 2012). TJ proteins with altered expression or function affect the tightness of epithelial surfaces, causing BBB hyper-permeability and consequently vasogenic oedema (Figure 1). It has been shown that occludin is downregulated and subsequently phosphorylated in human high-grade gliomas, thus causing increased permeability of the TJs as a result of the altered interaction between phosphorylated occludin and ZO1, ZO2 and ZO3 (Rubin and Staddon, 1999; Papadopoulos et al., 2001; Kale et al., 2003). Liebner et al. (2000) have shown that the expression of the TJ protein claudin 1 is lost in the majority of tumor micro vessels and claudin 5 down-regulated in hyperplastic vessels, leading to increased endothelial permeability. GBM cells secrete several factors including VEGF, stromal cell-derived factor-1 (SDF-1α) and Angiopoietin 1 (Ang-1) that increase the proliferation of endothelial cells and promote development of new vessels. To further aggravate the condition, the tumor’s vascularization is immature and morphologically abnormal. Indeed, the new vessels are disorganized, deformed, tortuous, partially occluded, excessively leaky and dysfunctional (Hardee and Zagzag, 2012; Dubois et al., 2014; Salmaggi et al., 2004). The main factor that promotes neo-angiogenesis is VEGF, which is up-regulated in brain tumors associated with oedema (Lin and Wang, 2016; Lin et al., 2016). VEGF affects the vascular endothelium, stimulating the proliferation and migration of endothelial cells, decreasing the expression of TJ proteins, whilst increasing the hydraulic permeability of vessels (Hardee and Zagzag, 2012; Stokum et al., 2016). Overall, altered TJs in endothelial cells and abnormal vascularization result in fluid buildup in cerebral parenchyma, leading to increased brain volume and ICP (Papadopoulos et al., 2004).

## Management of Brain Oedema With DEX

GCs are steroid hormones produced by the adrenal cortex under the control of the hypothalamic–pituitary–adrenal axis (HPA) that regulate carbohydrate metabolism, inflammatory and immune responses. Their effects are mediated by binding to GC receptor (GR) on a time scale from hours to days (genomic effects). The best known and well characterized isoform of GR is GR-α. The complex GC-GR binds to DNA at particular glucocorticoid responsive elements (GRE) and regulates the transcription of a number of genes (Piette et al., 2006).

GCs reduce oedema by affecting BBB functionality, particularly through modulation of gene expression and function of claudins, occludins, and vascular endothelial (VE)-cadherin that regulate endothelial permeability (Hue et al., 2015). In rodent brain tumors, DEX decreases BBB permeability by up-regulating the expression of occludin (Gu et al., 2009a,b).

**Figure 2:**
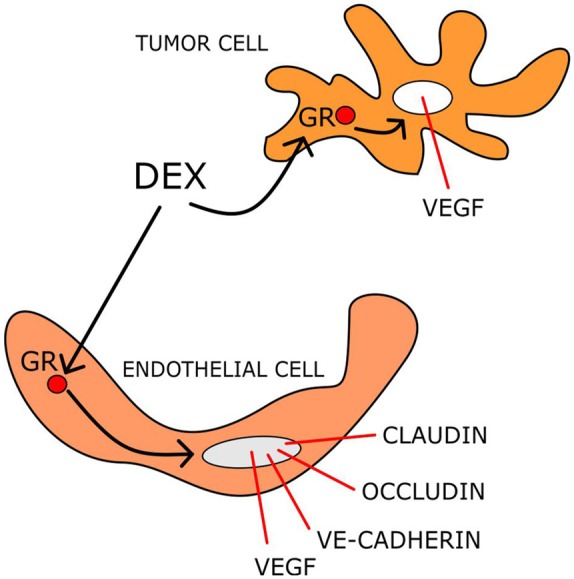
DEX mechanisms reducing tumor-induced brain oedema. DEX acting on both endothelial and tumor cells modulates the transcription of several gene products that control the BBB permeability.

The stability of the BBB is regulated by Ang-1 and Ang-2, as well as VEGF (Kaal and Vecht, 2004; Nag et al., 2005). Ang-1 binds to the receptor tyrosine kinase Tie-2 expressed on endothelial cells, whereas, Ang-2 participates in BBB breakdown through vessel destabilization (Maisonpierre et al., 1997). Up-regulation of Ang-1 and down-regulation of VEGF by DEX treatment have been reported in human brain astrocytes and pericytes (Kim et al., 2008). DEX ameliorates oedema not only by influencing the factors that regulate the permeability of the capillary bed, but also by inhibiting their production (Figure 2). Indeed in C6 cells, DEX down-regulates VEGF mRNA and protein expression in a dose-dependent manner both in normoxic and hypoxic conditions. This effect was found to be mediated by GR (Finkenzeller et al., 1995; Nauck et al., 1998; Machein et al., 1999).

The ability of DEX to modulate the expression of several potassium channel types in the BBB of glioma has also been demonstrated (Zhang et al., 2007). In particular, DEX treatment increased the expression of calcium-activated K^+^ channels in the BBB of rat brain glioma (Gu et al., 2007; Gu et al., 2009b). In the same glioma model, up-regulation of ATP sensitive-K^+^ channel (K_ATP_) expression by DEX has also been reported (Gu et al., 2007). Through up-regulation of these K^+^ channel types, DEX could regulate transcellular pathways of brain tumor microvessels controlling BBB permeability. Although DEX seems to reduce oedema formation by modifying the permeability of capillaries within the tumor and enhancing the clearance of extracellular water (Swaroops et al., 2001; Sinha et al., 2004), the mechanisms of its actions are as to date not fully understood.

## GBM-Induced Abnormal Cell-Proliferation

Cell proliferation is a physiological process that involves the activation of many signaling pathways. GBM and several genetic abnormalities associated with this cancer alter various signaling pathways that control cell proliferation.

EGFR plays an important role in cell proliferation principally through receptor-mediated activation of PI3K/AKT/mTOR and GRB2/MEK/ERK/MAPK signaling pathways (Chistiakov et al., 2017). Amplification or mutations of EGFR are frequently found in GBM (Maire and Ligon, 2014). Indeed, 30% of GBM cases express the truncated mutant EGFRvIII receptor that is deprived from its extracellular ligand binding site and as a consequence is activated, constitutively (Frederick et al., 2000; Paff et al., 2014). By stimulating the downstream signal transduction pathways promotes tumorigenicity (Wang et al., 2009; Chistiakov et al., 2017). Importantly, the genetic analysis of EGFR in GBM is widely used as a diagnostic biomarker (Maire and Ligon, 2014).

*Phosphatase and tensin homolog* (PTEN) is a tumor suppressor that dephosphorylates the second messenger phosphatidylinositol 3, 4, 5-triphosphate, inhibits the PI3K/AKT pathway and reduces cyclin D1 accumulation, inducing cell cycle arrest at the G_0_/G_1_ phase. Mutations in PTEN are found in 20%–40% of cases that are mainly affected by primary GBM. Loss of function mutations in PTEN activate signaling molecules including phosphatidylinositol-dependent kinases (PDKs), serine/threonine kinases, AKT/protein kinase B, S6 kinase, and mTOR, as well as the small GTPases Rac1 and Cdc42 (Alexiou and Voulgaris, 2010). The overall effects resulting from activation of these signaling pathways are increased cell proliferation and tumor growth. In addition to cell proliferation, the PI3K/Akt/mTOR pathway regulates apoptosis and angiogenesis. Notably, this pathway is up-regulated in GBM and mediates uncontrolled cell proliferation, tumor growth and multidrug resistance (Zhao et al., 2017). As such, intense investigations are aimed to identify therapeutic strategies to inhibit the PI3K/AKT/mTOR pathway (Ströbele et al., 2015; Li et al., 2016).

Recent studies, have highlighted the important role of ion channels in GBM cell proliferation and aggressiveness (Sforna et al., 2017; Wong et al., 2018; Yang et al., 2017, 2018; D’Alessandro et al., 2018). In particular, it has been shown that the *swelling-activated Cl^−^ current* is over-expressed in GBM cell lines (Catacuzzeno et al., 2014; Sforna et al., 2017), and contributes substantially to cell proliferation through activation of JAK2/STAT3 and PI3K/AKT signaling pathways (Wong et al., 2018). Indeed, the inhibition of this Cl^−^ current reduced cell viability, proliferation and migration. However, cell volume changes are detected by the *Volume Regulated Anion Channel* (VRAC) that is composed of members of the *leucine-rich repeat-containing protein 8* (LRRC8) gene family, LRRC8A–E (Qiu et al., 2014; Voss et al., 2014). Downregulation of LRRC8A expression reduces GBM cell proliferation and increases sensitivity to the clinically used Temozolomide (TMZ) and Carmustine (Rubino et al., 2018).

## The Anti- and Pro-Proliferative Effects of DEX

The effects of DEX on GBM cell proliferation depend on cell type, drug concentration and experimental conditions (Piette et al., 2006). The anti-proliferative effect of DEX has been shown in several cell lines (e.g., T98G, A172, 86HG39, F98, GL261, U87; Kaup et al., 2001; Fan et al., 2014). By contrast, Fan et al. (2014) found that DEX did not compromise the vitality of T98G and U251 cells. However, they showed that DEX significantly decreased the percentage of F98 cells in the S-phase favoring the G_0_/G_1_ phase of cell cycle, thus highlighting its cytostatic effect (Fan et al., 2014). Piette et al. (2009) reported that DEX attenuated cell proliferation, through inhibition of the ERK1/2 MAPK pathway. It has also been shown that DEX interrupted cell cycle progression through down-regulation of cyclin D1 and inhibition of ERK1/2 phosphorylation, without altering the overall expression level of ERK1/2 (Liu et al., 2009). Necrotic death of C6 cells can be induced by serum deprivation and DEX enhanced this cytotoxic effect through GR activation (Morita et al., 1999). In the same cell line, the anti-proliferative effect of DEX was shown to be dose and time dependent and to involve Aquaporin 1 (AQP_1_; Guan et al., 2018). Indeed, DEX increased the mRNA and protein expression of AQP_1_. Moreover, the effect of DEX on cell proliferation was abolished upon AQP_1_ gene silencing (Guan et al., 2018). Surprisingly, by using U373 cells, Gündisch et al. (2012) showed a pro-proliferative effect of DEX (Table 1).

**Table 1:** Effects of DEX on cell proliferation of glioma cell lines.

Cell line	DEX dose	Proliferation inhibition	References
A172 T98G 86HG39	5 nM– 0.5 μM	YES	Kaup et al. (2001)
F98 GL261 U87	1–200 μg/ml	YES	Fan et al. (2014)
T98G U251	1–200 μg/ml	NO	Fan et al. (2014)
U373	0.1 μM	NO	Gündisch et al. (2012)
U373	10 μM	YES	Piette et al. (2009)
C6	0.01 μM	YES	Guan et al. (2018)
C6	0.1 μM	YES	Liu et al. (2009)
GL261	1 μg/ml	NO	Villeneuve et al. (2008)

In murine xenograph models, *in vivo*, DEX reduced tumor mass (Villeneuve et al., 2008). GL261 cells were implanted in the brains of male C57BL/6 mice and DEX treatment (1 mg/kg) was administered twice daily. After 20 days of treatment, this resulted in a 33% reduction in glioma volume (Villeneuve et al., 2008). Although, DEX did not influence the *in vitro* proliferation of GL261 cells, the decrease in tumor mass could be accounted for its effects on Ang-2 expression (Villeneuve et al., 2008).

DEX increases the chemotherapeutic action of several drugs. Indeed, in a human glioma U87 xenograph model, DEX significantly increased the therapeutic effects of carboplatin/gemcitabine combined therapy (Wang et al., 2004). However, the opposite effect of DEX has also been observed, *in vitro*. In particular, the strong apoptotic effect of TMZ in T98G and U87 cells was antagonized by DEX pre-treatment (Das et al., 2004, 2008; Sur et al., 2005). Notably, DEX administration pre-RT reduced the survival of glioma-bearing mice (Pitter et al., 2016).

## GBM-Induced Cell Migration

The ability of GBM cells to migrate and invade healthy brain tissue is remarkable, and makes GBM cells excellent experimental models for studying migratory processes. Cell invasion comprises four basic steps: detachment of invasive cells from the primary tumor mass, adhesion to the extracellular matrix (ECM), matrix degradation and cell migration (Onishi et al., 2011). Matrix metalloproteinases (MMPs) have been implicated in the degradation of ECM in various physiological and pathological conditions. They are believed to be important factors in tumor invasion through their effects on components of the ECM (Nakada et al., 2003). A decisive role in glioma invasion is played by the gelatinases MMP-2 and MMP-9 and increased expression levels of MMP-2 is associated with glioma invasiveness (Rao, 2003).

Full surgical resection of GBM is impossible as cancer cells migrate actively along brain structures, including white matter tracks, interstitial space of the brain parenchyma, blood vessels and the subarachnoid space (Bellail et al., 2004; Cuddapah et al., 2014). Migration also requires physical contacts that the GBM cells establish with molecules of the vascular walls. It has been shown that bradykinin induces Ca^2+^ release from intracellular stores of GBM cells, promoting cell migration (Montana and Sontheimer, 2011). In addition, GBM cells release large amounts of glutamate in the brain parenchyma that kill neurons effectively and acts as an autocrine and paracrine signal to promote migration of cancer cells by triggering cytoplasmic Ca^2+^ oscillations (Ye and Sontheimer, 1999;Lyons et al., 2007).

To assume their characteristic migratory shape, GBM cells undergo cell volume changes and cytoskeletal remodeling. These processes are strictly dependent on the activity of several ion channel types. Indeed, Cl^−^ and K^+^ channels are over-expressed in GBM cells and facilitate their migratory features. The Cl^−^ and K^+^ ion flux through chloride channels and Ca^2+^-activated K^+^ channels (K_Ca1.1_ and K_Ca3.1_) allows cell volume changes that are essential events for effective GBM cell migration (Turner and Sontheimer, 2014; Catacuzzeno et al., 2015; Rosa et al., 2017). ClC-3 expression is up-regulated in glioma and correlates with WHO histological grade (Wang et al., 2017). Previous studies have shown the important role of ClC-3 in human glioma cells invasion through the application of pharmacological inhibitors or ClC-3 siRNA transfection (Lui et al., 2010; Cuddapah and Sontheimer, 2010). Recently, Wang et al. (2017) reported that knock-down of ClC-3, by using recombinant adenovirus expressing short hairpin RNA targeting human ClC-3 gene, inhibited the migration of U87MG cells significantly and reduced volume-regulated chloride currents in U87MG and SNB19 cells.

Previous studies have shown that hypoxia promotes GBM cell migration and increases tumor aggressiveness (Jensen, 2009; Yang et al., 2012). Extensive hypoxic areas are found within GBM mass that distinguish this tumor from those of low-grade malignancy. A distinctive histological pattern observed within the GBM is the palisade characterized by the presence of occluded vessels, around an extensive necrotic area and migrating cancerous cells (Rong et al., 2006; Amberger-Murphy, 2009). Sforna et al. (2017) reported that the *swelling-activated Cl^−^ current* is up-regulated during hypoxia, an effect that further facilitates GBM cell migration and brain infiltration (Wong et al., 2018).

## Effects of DEX on Cell Migration

Scant and inconsistent information is available on the effects of DEX on cell migration and this varies with the cell type under investigation. Inhibitory effects of DEX in migration/invasion of several glioma cell lines (e.g., C6, U251, U373, and A172), have been previously reported (Bauman et al., 1999). DEX inhibited the migration of U87 cells by reducing MMP-2 secretion (Lin et al., 2008). On the other hand, Piette et al. (2009) have shown that DEX treatment significantly reduced the migration and invasion of U373 cells through GR-dependent ERK1/2 MAPK pathway. It should be recalled that in GBM the ERK1/2 MAPK pathway is activated remarkably through EGFR and is linked to cell invasion and migration, as well as proliferation (Huang et al., 2009). Moreover, the isoforms p44 (ERK1) and p42 (ERK2) are stimulated by a wide variety of growth factors and mitogens (Johnson and Lapadat, 2002).

In stark contrast with previous studies, a recent report has shown that DEX facilitated C6 cells migration through up-regulation of AQP_1_ expression (Guan et al., 2018).

## DEX Therapy in GBM

Patients with primary brain tumors are commonly treated with DEX before and after biopsy or resection and during RT. Unfortunately, DEX causes many side effects including myopathy, abnormal glucose metabolism, gastrointestinal complications, irritability, anxiety, insomnia, and is linked to high risk of pneumonia infection (Kostaras et al., 2014). Although, myopathy is reversible upon DEX discontinuation, almost 50% of patients develop disturbed glucose metabolism which persists even after dose reduction (Wen et al., 2006). Most psychiatric complications occur within the 1st week of therapy. The severity of side effects increases with the dose regimen and length of treatment. Thus, it is recommended that DEX therapy be reduced once symptoms commence to improve (Kostaras et al., 2014).

Upon DEX treatment apoptosis of T cell is observed, accounting for its immunosuppressant effects (Dietrich et al., 2011). The potent immunosuppressive actions of GCs could raise the question as to whether these drugs could weaken the body immune response against cancer cells and contribute to the short survival of GBM-affected individuals. However, due to its immunosuppressant activity, DEX interfered with conventional chemotherapies or electric field-based therapy delivered by the NovoTTF-100A (Hughes et al., 2005; Grossman et al., 2011; Wong et al., 2015). This is a new medical device used for chronic treatment of patients with recurrent or progressive GBM that exploit alternating electric fields (termed TTFields).

A number of studies have demonstrated DEX effectiveness in management of patients with brain peri-tumor oedema by ameliorating the symptoms associated with vasogenic oedema and intracranial high pressure within 48 h of treatment (Galicich et al., 1961; Hossmann et al., 1983; Ostergaard et al., 1999; Sinha et al., 2004; Dietrich et al., 2011; Kostaras et al., 2014). An excellent technique for monitoring tumor-related oedema in patients is magnetic resonance imaging (MRI) that allows the evaluation of various water diffusion parameters (Yamasaki et al., 2012; Bode et al., 2006; Kural et al., 2018). Using diffusion tensor imaging, Sinha et al. (2004) examined individuals with intracranial tumors and observed a significant reduction in the mean diffusivity of brain oedema after 48–72 h post-DEX treatment. However, a recent study conducted in 28 patients with different degrees of glioma, brain metastases and neurological deficits, reported that DEX had no significant effect on the volume of peritumoral oedema in 19 patients, while some improvements were observed in the remaining (Kural et al., 2018). Given the very frequent use of DEX in GBM therapy and the controversies concerning its effects there is now a renewed interest to evaluate the correlation between the use of this drug and the overall survival (OS) rate of treated patients. A recent study showed that administration of DEX during RT in patients treated with TMZ, was a poor prognostic indicator of both OS and progression-free survival (PFS). Therefore, DEX treatment before or concomitantly with TMZ was not recommended. However, the OS and PFS were not affected by DEX in patients who received a combination therapy with both TMZ and Bevacizumab (BEV), during RT (Shields et al., 2015). Important observations about the use of DEX in GBM therapy have been provided by Wong et al. (2015). Indeed, they showed that patients treated with higher DEX doses (>4.1 mg daily) had significantly shorter OS than those treated with lower doses (<4.1 mg daily). DEX-treatment at high doses (6–16 mg/day) resulted in the disappearance of the tumor mass in some patients. Remarkably, the tumor reappeared after 1–4 weeks and was much more aggressive (Buxton et al., 1997; Zaki et al., 2004; Goh et al., 2009). It has also been observed that DEX therapy reduced the OS of a particular subgroup of GBM patients (Dubinski et al., 2018). In this study, 35 patients received preoperative DEX and compared to 78 control patients. The OS value was not different between these two main groups. However, within the subgroup of patients who received DEX, 22 individuals showed DEX-induced leukocytosis (DIL) and had shorter OS that those without DIL. As such, patients with DIL were considered at a high risk of poor outcome (Dubinski et al., 2018).

In patients with cancer, inter-individual differences in drug response influences both the outcome to therapy, as well as the prevalence of adverse effects. Along with the progress in diagnosis, based on tumor molecular characteristics, genomic and proteomic approaches continue to be developed to ameliorate individualized treatment (Shai et al., 2008). Changes in protein expression due to altered gene regulation in drug-target genes can affect the efficacy or toxicity of the drug. This concept applies especially to GCs, as well as to other drugs used in the management of GBM. Indeed, GCs are known to regulate the expression of hundreds of genes, whose dysregulation could favor a poor prognosis for GBM patients. In a study that analysed the type of genes affected by DEX treatment from patient-derived GSC lines several genes associated with cell proliferation and migration were either up-regulated or down-regulated by the drug (Luedi et al., 2017). Importantly, DEX-responsive genes were found to be prognostic of poor outcome in two independent GBM cohorts (Luedi et al., 2017). Both, *in vitro* and *in vivo* evaluation of DEX effects along with the analysis of clinical information from *The Cancer Genome Atlas* (TCGA) cohort, important correlations have been derived between DEX treatment and alterations in gene expression profiles. The comparison between patients with mesenchymal and proneural GBM showed that the former subgroup had significant up-regulation of DEX-controlled gene network as well as pathways closely related to proliferation, invasion, and angiogenesis. The effects of DEX on invasion, proliferation and angiogenesis in GBM patient–derived GSCs with IDH1 wild-type (GSC3) and with IDH1 mutant (GSC6) were very recently evaluated. DEX significantly increased the invasive properties of the GSC3 cell line, both *in vitro* and *in vivo*. Moreover, using both GSC3 and GSC6 lines, the drug promoted proliferation and angiogenesis, *in vivo* (Luedi et al., 2018). The results of this interesting study, have questioned the use of DEX in GBM therapy, highlighting its ability to increase the aggressiveness of the tumor.

## Concluding Remarks

Since 1960, DEX represented the gold standard for treatment to reduce oedema caused by brain tumors, although current literature fails to clearly delineate the mechanism of action. In this review, we have discussed several critical factors regarding the influence of DEX in GBM. Overall, contradictory effects of DEX opposing or favoring GBM aggressiveness have emerged from a number of *in vitro*, *in vivo* and clinical studies. It has been argued that this conflicting data was a result of different experimental conditions (Mealey et al., 1971; Grasso et al., 1977; Pinski et al., 1993; Langeveld et al., 1992; Morita et al., 1999; Piette et al., 2006). Considering this variability and current controversial issues, investigators are focusing on genetic biomarkers and transcriptional signature that are influenced by DEX. Knowledge derived through these studies is highly needed to provide prediction in patient OS. The urge to evaluate other subtypes of GBM that are distinguished by different genetic and epigenetic alterations, the distinct susceptibility to DEX and their changes in the degree of malignancy upon drug exposure is inevitable in future studies. Indeed, establishing the GBM type that can or cannot be treated with DEX along with early diagnosis shall increase patients’ life expectancy, considerably.

*Pharmacogenomics* promise the advent of precision medicine. Thus, to optimize DEX therapy with respect to patients’ genotype and ensure maximum efficiency with minimal adverse effects, genome-wide association investigations incorporating genomics and epigenetic approaches, should be considered. The recognition of the various desirable and undesirable pathways that are activated in GBM by GCs could pave the way towards a combinational therapy, consisting in the co-administration of a classical GC with drugs that inhibit the unwanted pathways activated by that specific GC. Furthermore, a road map guiding progress towards development of selective GR agonists and modulators with a more restricted GR activity, which retain efficacy without eliciting unwanted adverse effects should be established (Sundahl et al., 2016). Overall, these strategies could settle the “*DEX issue in GBM*” and allow the prescription of this drug to distinct subsets of patients or through individual therapy depending on one’s genetic makeup.

## Author Contributions

MC, MV, SB, MD and MP contributed to writing the manuscript. LS, PR and SR contributed to revising the manuscript. MP coordinated the writing, revised and submitted the manuscript.

## Conflict of Interest Statement

The authors declare that the research was conducted in the absence of any commercial or financial relationships that could be construed as a potential conflict of interest.
